# Correlation AnalyzeR: functional predictions from gene co-expression correlations

**DOI:** 10.1186/s12859-021-04130-7

**Published:** 2021-04-20

**Authors:** Henry E. Miller, Alexander J. R. Bishop

**Affiliations:** 1 Greehey Children’s Cancer Research Institute, University of Texas Health At San Antonio, San Antonio, TX 78229 USA; 2 Department of Cell Systems and Anatomy, University of Texas Health At San Antonio, San Antonio, TX 78229 USA; 3 Mays Cancer Center, University of Texas Health At San Antonio, San Antonio, TX 78229 USA

**Keywords:** Co-expression, RNA-Seq, Data mining, Systems biology, R shiny, Web application, Gene function, Gene correlation

## Abstract

**Background:**

Co-expression correlations provide the ability to predict gene functionality within specific biological contexts, such as different tissue and disease conditions. However, current gene co-expression databases generally do not consider biological context. In addition, these tools often implement a limited range of unsophisticated analysis approaches, diminishing their utility for exploring gene functionality and gene relationships. Furthermore, they typically do not provide the summary visualizations necessary to communicate these results, posing a significant barrier to their utilization by biologists without computational skills.

**Results:**

We present Correlation AnalyzeR, a user-friendly web interface for exploring co-expression correlations and predicting gene functions, gene–gene relationships, and gene set topology. Correlation AnalyzeR provides flexible access to its database of tissue and disease-specific (cancer vs normal) genome-wide co-expression correlations, and it also implements a suite of sophisticated computational tools for generating functional predictions with user-friendly visualizations. In the usage example provided here, we explore the role of *BRCA1-NRF2* interplay in the context of bone cancer, demonstrating how Correlation AnalyzeR can be effectively implemented to generate and support novel hypotheses.

**Conclusions:**

Correlation AnalyzeR facilitates the exploration of poorly characterized genes and gene relationships to reveal novel biological insights. The database and all analysis methods can be accessed as a web application at https://correlationanalyzer.bishop-lab.com/ and as a standalone R package at https://github.com/Bishop-Laboratory/correlationAnalyzeR.

**Supplementary Information:**

The online version contains supplementary material available at 10.1186/s12859-021-04130-7.

## Background

Almost two decades after the completion of the Human Genome Project, the functionality of many genes remains largely enigmatic [1]. Many such “enigmatic genes” have immense biological significance, exemplified by the associations of thousands with cancer outcome [2]. Even genes which are well-characterized often play unexpected roles in different biological contexts (e.g., *EZH2* is both a tumor-suppressor and an oncogene in different cancers [3]). Gene co-expression correlations provide a robust methodology for predicting gene function, as genes which share a biological process are often co-regulated [4–6]. Similar insights can be gained from using protein interaction (for example STRING [7] and InterologFinder [8]), phenome data, or even the combination of both [9]. Irrespective, generating expression data remains a cost-effective approach and co-expression analysis remains a prominent tool for exploratory systemic evaluation, largely because it is capable of considering gene co-expression across the genome. However, the applications which have been developed for such inference are hampered by key limitations. Tools like COXPRESdb [10] and GeneFriends [11] calculate gene set over-representation on an arbitrary number of co-expressed genes. Alternatively, GeneMANIA [12] and GIANT [13] construct co-expression networks and calculate gene set over-representation on an arbitrary number of nodes. Neither approach is sensitive to the genome-wide distribution of co-expression correlations or, with the exception of GIANT, differences between tissue/disease conditions. Furthermore, these functional predictions are limited in scope and do not generate relevant, user-friendly visualizations, limiting their utility for biologists without bioinformatics skills.

Recently, Lachmann et al. introduced ARCHS4, a database with thousands of standardized RNA-Seq datasets [14]. We re-processed these data, calculating co-expression correlations with respect to tissue and disease (cancer/normal) condition and provided the results in a publicly accessible database. We now present *Correlation AnalyzeR*, a user-friendly interface to this co-expression database with a suite of tools for *de-novo* prediction of gene function, gene–gene relationships, and biologically relevant gene subgroups to facilitate discovery of novel relationships within genes of interest.

## Construction and content

### Code availability

We have provided the source code for the *correlationAnalyzeR* R package and the *Correlation AnalyzeR* web application for public use. All preprocessing scripts, custom regex dictionaries, and scripts for generating any figures not generated by Correlation AnalyzeR can be found in the *misc/* directory of the correlationAnalyzeR repository. Github repositories: [R-package: https://github.com/Bishop-Laboratory/correlationAnalyzeR, Shiny app: https://github.com/Bishop-Laboratory/correlationAnalyzeR-ShinyApp].

### ARCHS4 data source

Re-processed RNA-Sequencing counts were generated by the authors of the ARCHS4 repository [14]. This data source comprises standardized counts across 238,522 human sequencing samples (ARCHS4 v8, Feb 2020) generated from the Illumina HiSeq 2000, HiSeq 2500, or NextSeq 500 platforms. Each sample has a corresponding entry in the Gene Expression Omnibus (GEO) database, complete with a tissue description, among other metadata categories.

### Generation of tissue- and disease-specific correlation data from RNA-Seq counts

We downloaded read counts and metadata from ARCHS4 and pre-processed them with custom R scripts in multiple stages:

1. Categorization
2. Filtering
3. Normalization, transformation, and correlation calculation

We have provided final correlation matrices via the *getCorrelationData* function in the correlationAnalyzeR R package, through the Correlation AnalyzeR web interface, or through SQL query using the access credentials provided in the *getCorrelationData* function source code.

### Sample categorization

We categorized samples based on their tissue descriptions from GEO, using a manually curated regex dictionary in conjunction with the Cellosaurus ontology [15]. For example, the terms “^pfc$” and “\bstriatum\b” positively identify brain samples with high specificity. However, we also implemented a logical matching system that could rule out an incorrect mapping which arises from term ambiguity. For example, brain samples are positively identified by “cortex”. However, since this could also refer to the kidney, brain samples are also negatively matched to the “kidney” term. This means that the brain label might be applied if “cortex”, but not “kidney”, is found in that sample’s tissue description in the GEO database. Category assignments were checked for sanity via manual inspection of randomized samples and the regex dictionary was adjusted accordingly when mistakes were noted. We assigned sample disease status using a manually curated dictionary of disease-related regex terms. Cancer, but not other diseases, was readily identifiable from available sample metadata, allowing classification of samples into *cancer* and *normal* (non-cancer) groups.

### Dataset filtering

We filtered the read count data using a three-step procedure:

1. Single cell RNA-Seq (scRNA-Seq) samples were identified from the GEO metadata using a custom regex dictionary and removed because of the demonstrated unsuitability of single cell data for co-expression network inference by Pearson correlation [16].
2. Samples with fewer than 5 million raw read counts were discarded to improve the quality of gene co-expression calculations by reducing noise from low-quality samples [17].
3. Tissue-disease groups with fewer than 30 distinct samples were removed to limit the effects of bias from individual samples and improve the performance of co-expression calculations [17].

The final set of filtered and labeled samples along with original tissue descriptions from GEO, number of reads aligned to the genome, GEO sample IDs, and GEO Series IDs is provided here (Additional file 2: Table S1).

### Normalization, transformation, and correlation calculation

We normalized and transformed the count data in a single step using the *vst* function of the *DESeq2* R package [18]. This function first calculates sample geometric means, estimates dispersions for each gene, fits a mean-dispersion trend, and then transforms the data to make it homoscedastic. Then, we calculated gene–gene Pearson correlations separately for each tissue-disease group using the *cor* function of the *WGCNA* package [19]. Each correlation matrix row was subsequently transformed into a string of comma-separated values, uploaded to an Azure MySQL server, and indexed for rapid query. The VST counts were also uploaded to the MySQL database.

### Comparison of Pearson and Spearman correlation methods

Due to the assumptions made by Pearson correlation about linear relationships between continuous features, it was important to compare it to a correlation method that can find non-linear, monotonic relationships, for which the Spearman method was a logical choice. To test these correlation methods, we obtained the “Hallmark” collection from the Molecular Signatures Database (MSigDB) v7.2 [20–22], via the *msigdbr* R package [23]. This collection was curated using a combination of unsupervised learning, manual curation, and independent validation [20]. The gene sets in this database are known to be both co-expressed and functionally related [20], indicating that a suitable co-expression metric for Correlation AnalyzeR should be capable of recognizing them as correlated. Then, we performed a permutation experiment with 2832 simulations in which each involved randomly selecting a “Hallmark” gene set and then randomly selecting a gene pair within that gene set. For each selected gene pair, the Pearson and Spearman correlation was calculated using the VST-transformed expression values for those genes and the *cor* function. The distribution of correlation values was compared using a one-tailed t-test based on the hypothesis that Pearson would prove more effective. We also used the difference between the Pearson and Spearman coefficients to identify the top Pearson-specific and Spearman-specific gene pairs. The results of this analysis are found in the *Choice of Pearson correlation* subsection of the *Discussion*.

### Validation of correlation values with external databases

To validate our correlation values, we compared them to ARCHS4 [14], COXPRESdb [24], and GeneFriends [11]. For three genes (*BRCA1*, *AURKB*, and *HSP90AA1*), genome-wide correlations were downloaded from each service. Of note, we derived the Correlation AnalyzeR correlations from all tissues and disease conditions so that they would be comparable with these external databases. We calculated and visualized the Spearman correlation between databases using the *PerformanceAnalytics* R package [25]. The top 500 correlations for each gene were also compared to the list of protein interactors for that gene (BioGRID) [26]. We calculated the overlap between correlations and protein interactors, assessing significance with the hypergeometric test from the R *phyper* function and visualizing the overlap using the *VennDiagram* R package [27]. The results of this analysis are found in the *Comparison with existing datasets* subsection of the *Discussion*.

### Correlation AnalyzeR

The *Correlation AnalyzeR* application is written in the R language [28] and can be accessed conveniently through a user-friendly web interface written in *R-Shiny* [29] [http://gccri.bishop-lab.uthscsa.edu/correlation-analyzer/] or installed locally as an R package [https://github.com/Bishop-Laboratory/correlationAnalyzeR]. Correlation AnalyzeR contains methods for retrieving tissue- and disease-specific co-expression correlations and implements four main modes of analysis:

1. Single gene
2. Gene versus gene
3. Gene versus gene list
4. Gene list topology

### Single gene

*Single gene* mode (called by the *analyzeSingleGenes* function within the R-package implementation) is the cornerstone of the Correlation AnalyzeR approach to gene function prediction. For any gene of interest in any tissue-disease group, this method rapidly retrieves genome-wide correlations and infers gene function using a custom implementation of *Gene Set Enrichment Analysis* (GSEA) [21]. GSEA is classically utilized to determine the top differentially expressed pathways between two conditions. Our approach, termed *corGSEA*, leverages genome-wide Pearson correlations as a ranking metric for the GSEA algorithm to determine the gene sets correlated with a gene of interest. Of note, the implementation of GSEA used here is the “pre-ranked” method [21]. The results of corGSEA provide novel insights into the functions of a gene within various tissue and disease contexts. This approach also leverages a light-weight implementation of GSEA, *fGSEA*, that benefits from an approximate 10 × speed increase compared to the original algorithm [30]. The Molecular Signatures Database (MSigDB) v7.2 [20–22], accessed via the msigdbr R package [23], provides the annotations used by corGSEA. Finally, there is a second analysis mode within *Single gene* mode, called *Group mode*. In this mode, the correlations and expression levels for a single gene across all available tissue-disease groups are returned.

### Gene versus gene

*Gene versus gene* mode (called by the *analyzeGenePairs* function within the R-package implementation) predicts the differences between any two genes, tissue types, and cancer vs normal conditions using genome-wide Pearson correlations and corGSEA-derived gene set enrichment. For example, an analysis of two different genes with the same tissue or disease condition would proceed as follows: for each gene, genome-wide correlations are retrieved and corGSEA is calculated using the *analyzeSingleGenes* function. For each of the two genes queried, the variance between each of their gene correlations (Pearson’s R) is calculated (using the *rowVars* function from the *matrixStats* package [31]), thus revealing the top diverging co-expression correlations between the two genes of interest. The same approach is applied to find the top diverging corGSEA results between the two genes, using the variance of the normalized enrichment score (NES) as the metric. *Group* mode is also available in *Gene versus gene mode,* allowing users to return the gene versus gene correlations across tissue-disease groups (automatically applied when gene one is different from gene two), or cancer versus normal correlations across tissue groups (automatically applied when gene one is the same as gene two).

### Gene versus gene list

*Gene versus gene list* mode (called by the *geneVsGeneListAnalyze* function within the R-package implementation) compares a gene of interest to a list of secondary genes or an MSigDB gene set of interest to explore the degree of correlation between them. Significance testing uses a permutation t-test comparing the selected secondary genes to a list of random genes of the same size, implemented with the *boot* R package [32]. The p value distribution constructed during permutation testing approximates the likelihood that the primary gene and secondary gene list are correlated greater than would be predicted by random chance.

### Gene list topology

*Gene list topology* mode (called by the *analyzeGenesetTopology* function within the R-package implementation) provides a suite of tools for *de-novo* prediction of tissue- and disease-specific functional groups within a gene list or an MSigDB gene set using the co-expression correlations of each list member: (1) *Dimension reduction* analysis uses principal component analysis (PCA) as the input for hierarchical clustering with or without TSNE, implemented with the *Rtsne* R package [33]. This methodology implements dimensionality reduction using genome-wide correlations or the top 2500 correlations by variance if there are more than 100 genes in the input list. (2) *Variant genes* analysis identifies the top 1500 gene co-expression correlations by variance among the input gene list to construct hierarchical clustering. These clusters represent novel groupings within the original input list and identify the top co-expressions which contribute to their group identity. (3) *Pathway enrichment* analysis is a simple and convenient wrapper for the *clusterProfiler* [34] R package’s *enricher* function. The top enriched pathways in the input gene list are returned and visualized with the *dotplot* function of clusterProfiler. Of note, the web version of this function imposes a limit of 500 genes, but this limit is not present in the R package implementation.

### Implementation

Correlation AnalyzeR is implemented as a stand-alone R package [https://github.com/Bishop-Laboratory/correlationAnalyzeR], which is easily installed using the *devtools::install_github("Bishop-Laboratory/correlationAnalyzeR")* command, and as a user-friendly web application written in *R-Shiny*, accessible at [http://gccri.bishop-lab.uthscsa.edu/correlation-analyzer/]. Visualizations are generated using *ggpubr*, *ggplot2*, and *pheatmap* [35–37]. For the *shiny* implementation, interactive visualizations were generated using *plotly* and *heatmaply* [38, 39]. Interactive data tables were generated using the *DT* package [40]. The webserver is an Azure B4ms instance with 4 cores and 16 GB memory running Ubuntu 18.04 and the MySQL server is an Azure Basic, 1 vCore instance with 500 GB of storage.

## Utility and discussion

### Overview of correlation AnalyzeR

**Fig. 1:**
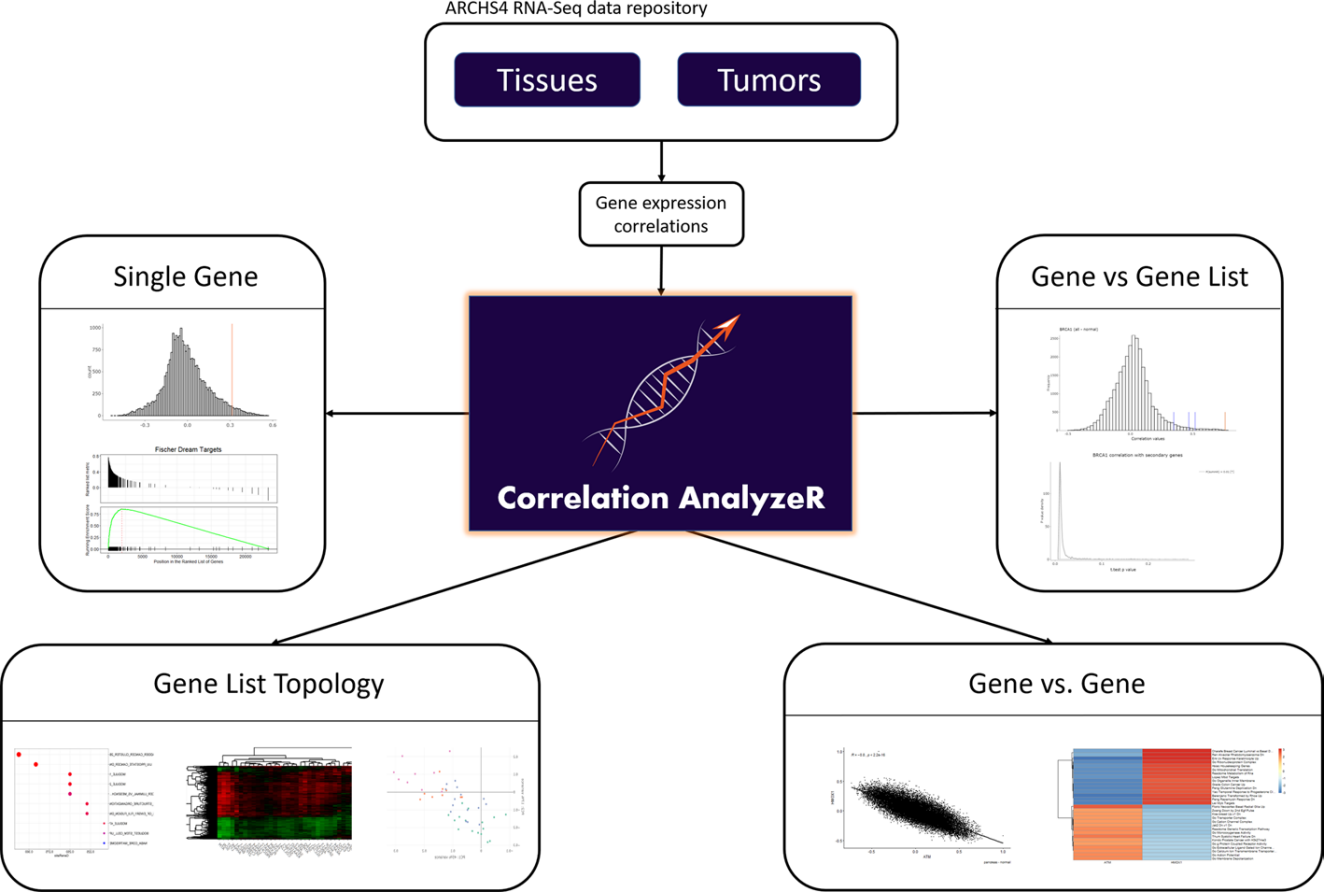
Overview of Correlation AnalyzeR. RNA-Seq read counts were preprocessed into gene co-expression correlations, which are analyzed in multiple analysis modes to yield novel biological insights

The *Correlation AnalyzeR* web application provides flexible access to the co-expression correlation database along with four main analysis modes which generate user-friendly visualizations and summary tables (Fig. 1): (1) Single gene, (2) Gene vs gene, (3) Gene vs gene list, and (4) Gene list topology.

*Single gene* mode returns co-expression correlations for a gene of interest and implements a unique procedure for predicting gene function using these correlations as the basis for *Gene Set Enrichment Analysis* (corGSEA). Visualized using *R shiny*, the result is a user-friendly interface for exploring genome-wide correlations with a gene of interest and its top correlated pathways. Within Single gene mode, *Group mode* is an option that allows users to explore a gene’s correlations across multiple tissue and disease types. *Gene vs gene* mode predicts the functional differences for a single gene between disease conditions (i.e. normal vs cancer) or tissue types (e.g. brain vs bone). It can also predict functional differences between two different genes (e.g. *ATM* vs *TP53*) or a single gene across different tissue/disease conditions. Using *Group mode* within gene vs gene, users can compare a single gene in normal and cancer conditions across tissue types, or they can compare two different genes in all tissue-disease conditions. *Gene vs gene list* mode allows the user to enter a primary gene and compare it to a list of secondary genes or an MSigDB gene set. *Topology* mode allows users to enter a list of genes or an MSigDB gene set and find *de-novo* sub-groups based on the topology of co-expression correlations.

To exemplify the utility of this database for enabling exploratory data analysis by biomedical researchers, we present here an illustrative analysis of *BRCA1*, a gene involved in transcriptional regulation and DNA repair [41], in the context of bone cancer. The plots and tables shown in these results were generated using Correlation AnalyzeR with only minor annotations in some cases.

### Usage example: exploring the BRCA1-deficiency phenotype of bone cancers

In a recent study, it was shown that hypertranscription leads to sequestration of *BRCA1* and decreased ability to perform DNA repair by homologous recombination in a pediatric bone cancer, Ewing sarcoma [42]. This leads to Ewing sarcoma having a BRCA-deficient-like phenotype, which is the reason these tumors are sensitive to PARP-inhibitors [42]. Interestingly, the most common bone cancer, osteosarcoma, also shows evidence of a similar BRCA-deficiency-like phenotype and PARP-inhibitor sensitivity [43, 44]. These findings indicate the importance of *BRCA1* in mediating bone cancer phenotypes; however, it is still unclear what the consequences of *BRCA1* deficiency are in these tumors, or how *BRCA1*-supported pathways (such as the *NRF2* pathway [45]) are impacted.

### Single gene mode

A primary area of research in the cancer field is understanding the way in which normal tissue processes are hijacked and dysregulated in a cancer context. Therefore, we began the present analysis by using the *Group Mode* feature of Single Gene mode to assess BRCA1 co-expression correlations across normal tissue types (Additional file 1: Figure S1). Interestingly, it was found that the highest expression of *BRCA1* occurs in *prenatal* tissues (e.g., embryos, morula, fetal tissues etc.) (Additional file 1: Figure S1A). This echoes previous findings which showed that *BRCA1* is an essential gene during many aspects of development [46]. Furthermore, the interactive heatmap provided by this analysis revealed the top 100 genes that are consistently co-correlated with *BRCA1* across tissue groups (Additional file 1: Figure S1B). When analyzed with gene set over-representation analysis, the co-correlated genes were revealed to belong to processes previously connected with *BRCA1* biology, including several pathways related to cell cycle progression and cancer [47] (Additional file 1: Figure S1C). Interestingly, the second most significant gene set was “Pujana Brca2 PCC Network”, a collection of genes which are co-expressed with BRCA2 [22]. This suggested a co-expression relationship between *BRCA1* and *BRCA2* in a normal tissue context. To further elucidate this relationship, we utilized the *analyzeGenePairs* function of the Correlation AnalyzeR R package to compare the expression of *BRCA1* and *BRCA2* across normal samples (Additional file 1: Figure S2B), finding that they are highly co-expressed. Additionally, given that *BRCA1* showed a consistent co-expression with genes related to cancer, it was unsurprising to find that *BRCA1* (and *BRCA2*) were both more highly correlated and more highly expressed in cancer samples when compared to normal samples (Additional file 1: Figure S2A, S2C).

**Fig. 2:**
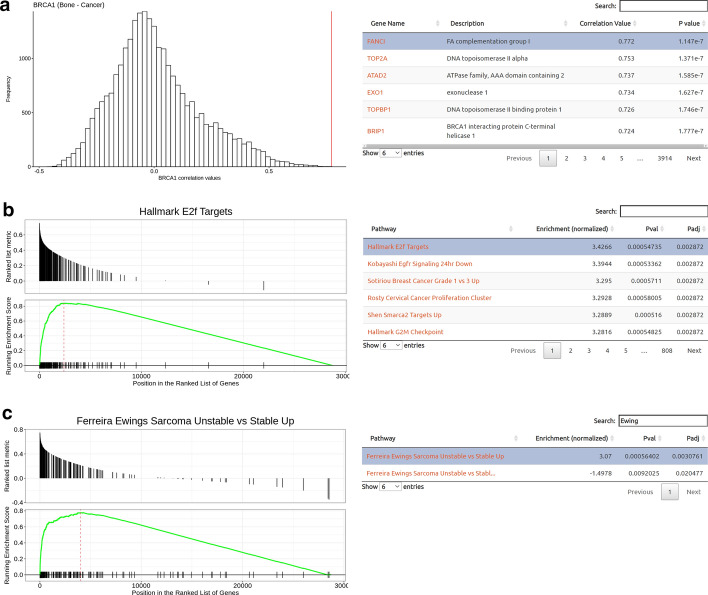
Analysis of BRCA1 in bone cancer tissue context using Correlation AnalyzeR single gene mode. **a** BRCA1 correlations in bone cancer samples represented as a histogram that can interactively indicate the position of a gene selected in the corresponding data table. P values are determined from the Pearson correlation coefficient. **b** corGSEA results for BRCA1 correlations represented. The visualization can interactively change when a user selects a new gene set. **c** corGSEA result for “Ferreira Ewings Sarcoma Unstable versus Stable Up” gene set. Search and selection features are demonstrated in the linked data table. **b**, **c** Enrichment, *p* values, and p adjusted values determined from GSEA algorithm (see "Methods" section)

To gain additional insight into the functionality of *BRCA1* in bone cancers, we used single gene mode to analyze the co-expression correlations in this tissue context (Fig. 2). The analysis results reveal the genome-wide co-expressions and their corGSEA enrichment within a user-friendly and interactive interface (Fig. 2). The interface displays a histogram of genome-wide correlation values (Pearson’s R and associated p value) with a linked data table for interactive exploration (Fig. 2a). The interface also shows the corGSEA results with a linked figure and searchable data table layout (Fig. 2b, c).

Interestingly, this analysis revealed the top co-expressed gene is *FANCI* (Fig. 2a), a gene which is over-expressed in Ewing sarcoma and which seems to play a role in preserving genome stability under conditions of hypertranscription [48]. However, no studies to date have examined a similar role for *FANCI* in other bone cancers, indicating a promising, unexplored line of experimental inquiry. Furthermore, the top *BRCA1* corGSEA results (Fig. 2b) predicted several well-established biological roles and relationships of *BRCA1*, such as its transcription by E2F transcription factors [49]. Interestingly, this analysis also suggested functionalities for BRCA1 (Fig. 2b) which have only been recently uncovered, such as its role in regulating *EGFR* signaling [50], but which have not yet been elucidated in the context of bone cancers. Finally, it was confirmed that expression of *BRCA1* in a bone cancer context is strongly correlated with markers of Ewing sarcoma genomic instability (“Ferreira Ewing Sarcoma Unstable vs Stable Up") [51] (Fig. 2c), as predicted by the previous evidence of Ewing sarcoma *BRCA1* sequestration [42].

In revisiting the Group Mode results, we examined the tissue-specific co-expression correlations of *BRCA1* (Additional file 1: Figure S3A) and observed an interesting tissue-specific relationship between *BRCA1* and *NQO1* (a target of *NRF2* and transcriptional proxy for *NRF2* activity [52]) (Additional file 1: Figure S3B). *BRCA1* shows a positive correlation with *NQO1* in some tissues, such as thyroid, but shows a negative correlation in others, such as stem-like tissues (Additional file 1: Figure S3B). Given the recently documented interplay between BRCA1 and NRF2 [45], the tissue-specific correlation between *BRCA1* and *NQO1* may indicate that this *BRCA1/NRF2* interplay is also tissue-specific. Furthermore, it indicates a potential consequence of *BRCA1*-deficiency in bone cancers in the differential regulation of the *NRF2* pathway, a pathway with important roles in antioxidant responses [53], drug resistance [54] and metastasis [55].

### Gene versus gene mode

**Fig. 3:**
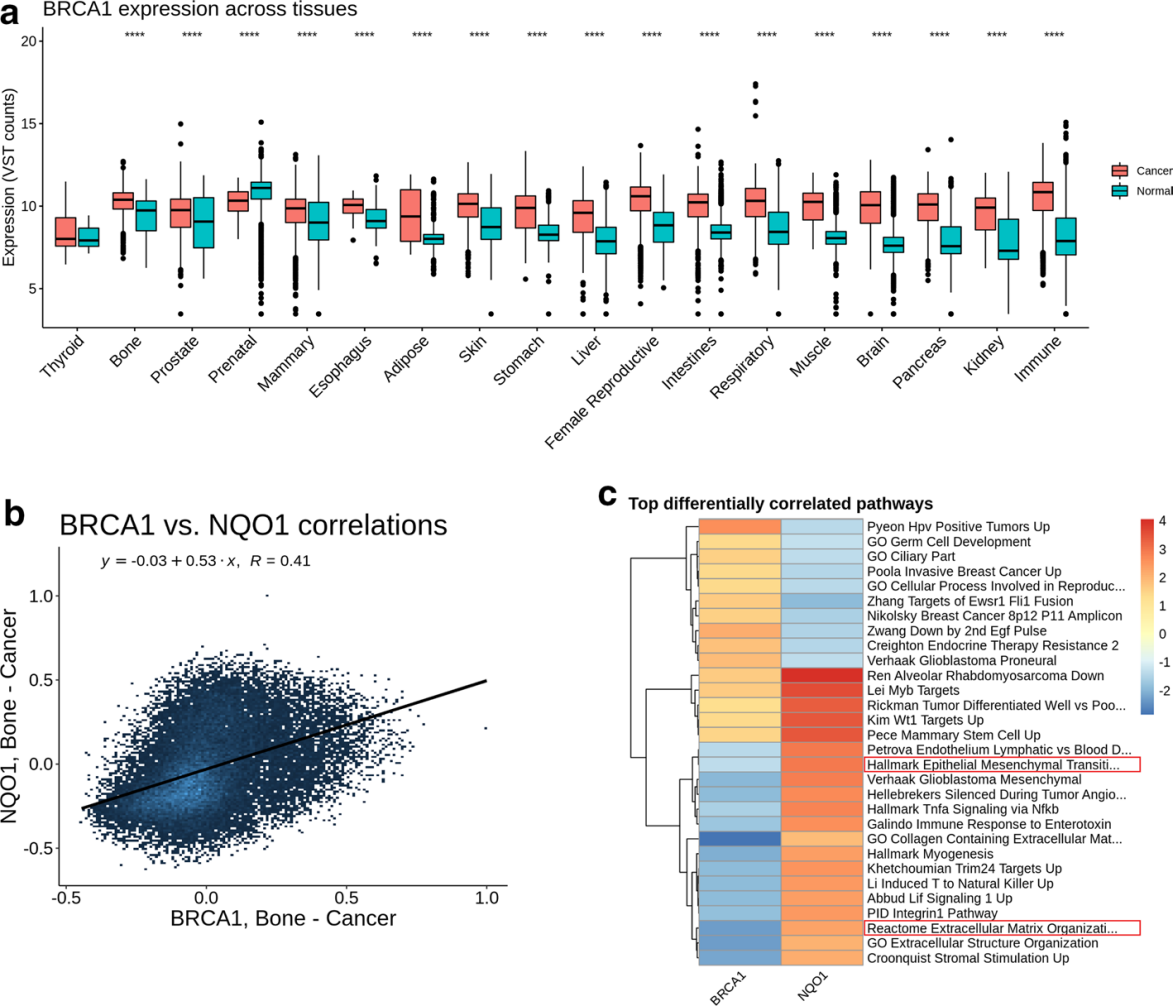
Analysis of BRCA1-NQO1 relationship with Correlation AnalyzeR Gene vs Gene mode. **a** Box plot showing difference between cancer and normal BRCA1 expression across tissues. Significance determined via the Wilcox rank sum test; *p < .05, **p < .01, ***p < .001, ****p < .0001. **b** Scatter plot showing the relationship between genome-wide co-expression correlations for BRCA1 and NQO1 in bone cancer tissues. Displayed R value determined by Pearson correlation. **c** Heatmap showing differential corGSEA results for BRCA1 and NQO1 in bone cancer tissues. Color bar displays the normalized enrichment score as determined by GSEA. Two cancer-related pathways are highlighted

The overexpression of *BRCA1* was previously demonstrated in two types of bone cancer, Ewing sarcoma [42] and osteosarcoma (Cancer Cell Line Encyclopedia; [56]). However, it is not known whether this association between *BRCA1* overexpression in cancer vs normal would be found for other tissue types. By using the Gene vs gene group mode, we found that *BRCA1* is overexpressed in cancer vs normal in most tissue contexts (Fig. 3a). Considering this result and our prior observation that *BRCA1* has a tissue-specific co-expression correlation with *NQO1* (Additional file 1: Figure S2B) we decided to further evaluate the *BRCA1-NQO1* relationship. We used gene vs gene mode to compare *BRCA1* and *NQO1* in bone cancer samples (Fig. 3b, c, and Additional file 1: Figure S4). We found that expression of *NQO1* and *BRCA1* displays both correlated co-expression and divergent expressions across different tissues, indicating the likely existence of important co-correlated and anti-correlated gene subgroups (Fig. 3b, Additional file 1: Figure S4A, and S4B).

Interestingly, by comparing the results of corGSEA between BRCA1 and NQO1, we found differential enrichment of several pathways implicated in cancer progression, such as “Hallmark Epithelial Mesenchymal Transition” and “Reactome Extracellular Matrix Organization” (Fig. 3c). This suggests the possibility that further dysregulation of these pathways may be associated with metastasis, indicating an interesting hypothesis which requires further wet lab analysis.

### Gene versus gene list mode

**Fig. 4:**
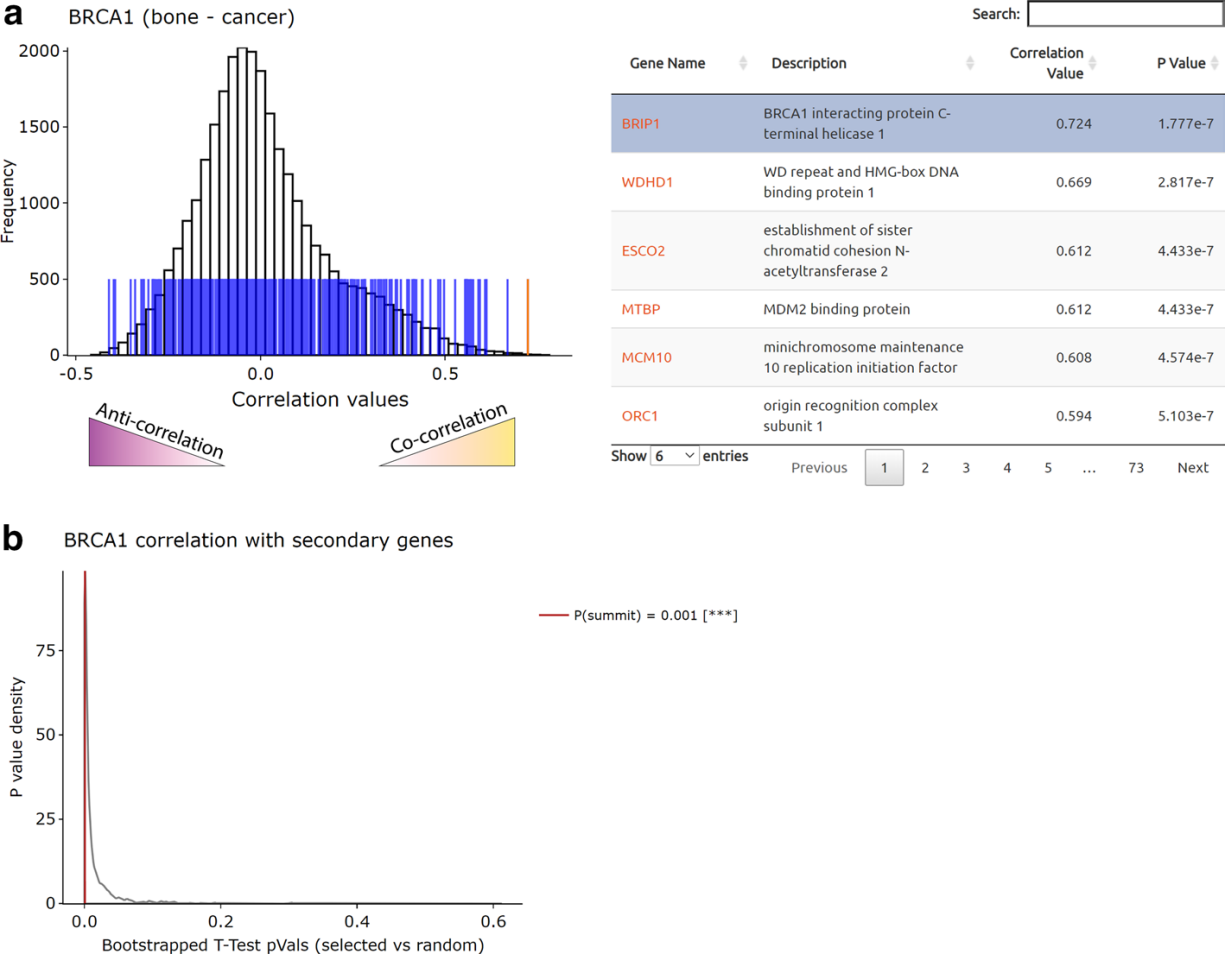
Analysis of BRCA1-NRF2 pathway interplay in bone cancer using Gene versus Gene List mode. **a**, **b** Results of comparing BRCA1 and NRF2 gene targets (“NFE2L2.V2” MSigDB gene set). **a** Interactive histogram showing the location of the NRF2-pathway genes (blue bars overlaid on histogram on left) within the correlation value distribution of BRCA1 with linked data table (right panel). P value determined from Pearson correlation. “Anti-correlation” and “Co-correlation” annotations not generated by Correlation AnalyzeR. **b** Density plot generated by permutation testing. The summit represents the empirically determined significance of BRCA1′s correlation with the NRF2 pathway (labeled “secondary genes” in plot) (see methods)

Using single gene and gene vs gene mode, we found a context-specific relationship between BRCA1 and the *NRF2* target *NQO1* (Additional file 1: Figure S3B) and that pathways related to cancer progression are differentially correlated between them in bone cancer (Fig. 3c). However, it was still unclear whether *BRCA1* is correlated with expression of the *NRF2* pathway in general or whether there are specific genes within this pathway beyond *NQO1* that are differentially associated with *BRCA1* expression. To address this question, we used gene vs gene list mode to compare *BRCA1* to the *NRF2* transcriptional targets MSigDB gene set (“NFE2L2.V2”; “*NFE2L2*” is the official gene symbol for *NRF2*) in bone cancer (Fig. 4). An interactive histogram was generated showing that *BRCA1* correlates positively with parts of the *NRF2* pathway, but negatively with others (Fig. 4a). Permutation testing was also conducted to generate an empirical p value distribution, demonstrating that the degree of correlation between *BRCA1* and the *NRF2* pathway in bone cancer is greater than would be predicted by random chance (Fig. 4b).

Interestingly, when we performed this analysis in the context of normal female reproductive tissues (e.g., ovaries, cervix), the correlation level was much more significant, with many strongly co- and anti-correlated genes (Additional file 1: Figure S5). Given that *BRCA1* mutations are associated with ovarian and breast cancer [57], the relationship observed with the *NRF2* pathway may suggest a role for those genes in normal tissue function and, possibly, cancer progression.

### Topology mode

**Fig. 5:**
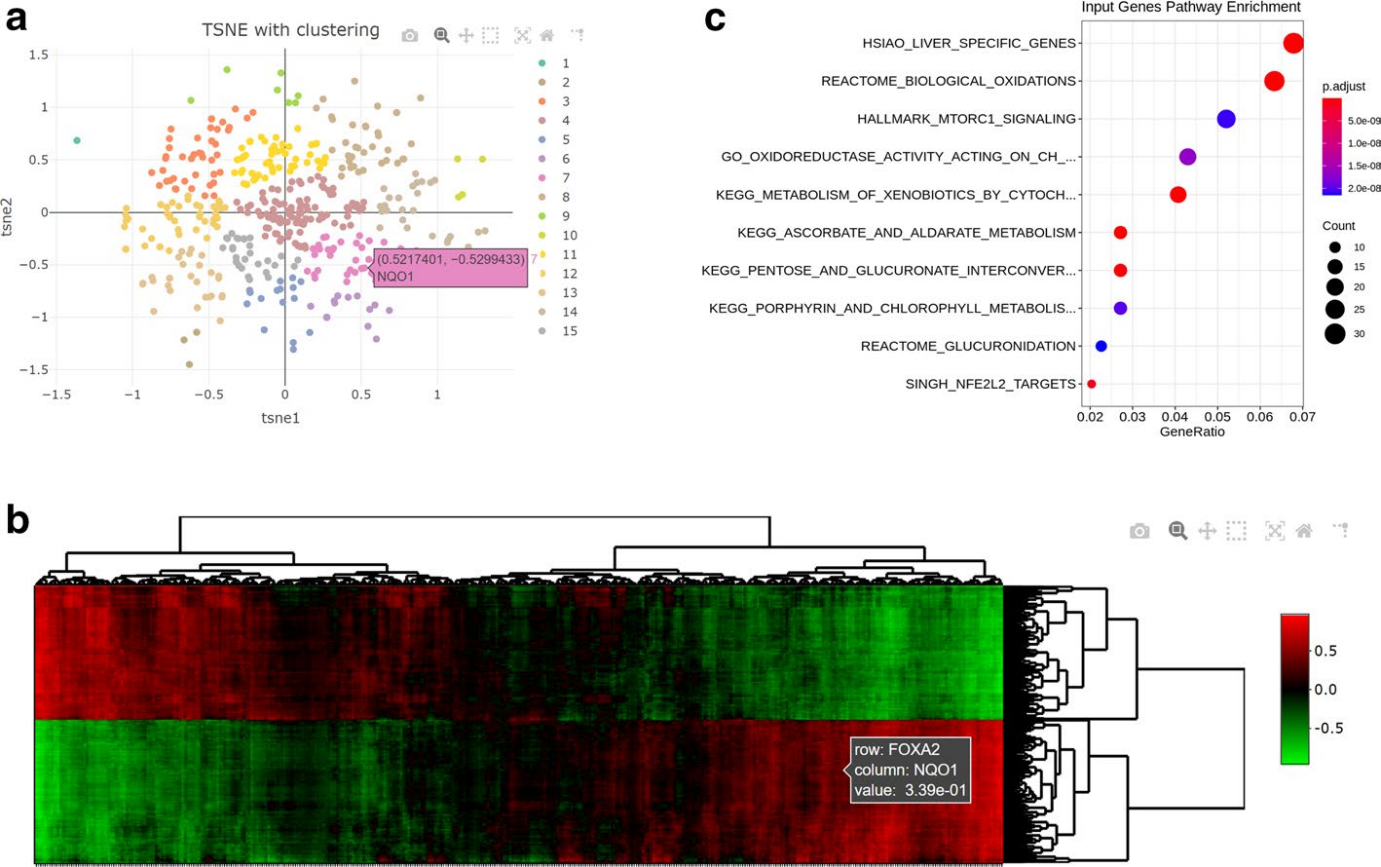
Analysis of the NRF2 pathway (“NFE2L2.V2” MSigDB gene set) in bone cancer using Topology mode. **a** Interactive TSNE plot representing each member of the NRF2 pathway with their de novo subgroups as determined by hierarchical clustering. The location of NQO1 within TSNE space is highlighted and is a component of cluster 7. **b** Interactive heatmap of the top 1500 correlations by variance between the members of the NRF2 pathway with NQO1′s position highlighted. Color bar represents Pearson correlation values. **c** Gene set over-representation analysis of the NRF2 pathway. P adjusted values calculated via Benjamini–Hochberg correction of over-representation *p* values

Having identified that some *NRF2* pathway genes correlate differentially with *BRCA1* in bone cancer samples (Fig. 4a), it was still unclear what subgroups of genes were represented by this difference. To address this question, we implemented topology mode using the *NRF2* targets MSigDB gene set (“NFE2L2.V2”) in bone cancer samples (Fig. 5). An interactive TSNE plot was generated showing the 2D embedding of this gene list with hierarchical clustering (Fig. 5a). By searching the interactive data table (not shown), we found that *NQO1* belonged to cluster #7, a cluster which included multiple other genes which share similar co-expression correlations in bone cancer. Additionally, an interactive heatmap was generated which displays the top 1500 co-expressions that account for variance within the *NRF2* targets gene set, revealing the genes which share similar and diverging co-expressions (Fig. 5b). Finally, the *NRF2* pathway genes were analyzed with pathway enrichment to determine what other gene sets are enriched in that gene list (Fig. 5c). As expected, oxidative stress response genes were uncovered along with genes related to glucuronidation (Fig. 5c).

## Discussion

By implementing the analysis modes provided by the *Correlation AnalyzeR* database application, we uncovered novel insights about *BRCA1′s* role in bone cancer, particularly with respect to the *NRF2* pathway. Furthermore, our analysis led to the identification of *NRF2* pathway members which might be involved in mediating bone cancer progression in response to changes in *BRCA1* activity. It remains for future wet lab research efforts to validate the mechanisms suggested by this exploratory analysis. All-in-all, the analysis provided here illustrates the power of *Correlation AnalyzeR* to leverage genome-wide co-expression correlations across diverging tissue contexts and provide novel biological insights.

In comparing Correlation AnalyzeR with similar tools (Additional file 3: Table S2), we find that it is a significant improvement over current methods. Not only does Correlation AnalyzeR offer a novel approach to gene function prediction (corGSEA), but it also provides multiple analysis modes not found in other tools, interactive plots that provide results in a user-friendly format, tissue and disease-specific analysis types, and an R package implementation for greater control over the analysis. However, it is also important to consider the quality considerations and practical limitations of this approach.

### Quality considerations for co-expression correlations

#### Dataset filtering

Several inherent challenges are posed when using RNA-Sequencing data for co-expression inference. Primarily, it is necessary to remove any relationship between read count variance and technical factors, such as library size. Therefore, our data processing approach included filtering out samples with terms such as “single cell”, “scRNA”, and “in-drop” in their descriptions, and samples with < 5 million reads. This filtering step was designed to mitigate the technical bias due to single-cell RNA-Sequencing and low-read-depth sequencing samples [16]. In manually checking 200 randomly selected samples from the filtered dataset, we were unable to find any instances of single-cell datasets, indicating the probable success of our approach.

#### Normalization and data transformation

Gene co-expression correlations are calculated from normalized datasets in which biologically relevant covariant relationships are preserved. Therefore, variance due to technical factors (e.g., library size) needs to be mitigated. We accomplished this by utilizing the median normalization approach implemented in DESeq2 [18]. Furthermore, a well-established phenomenon in RNA-Sequencing datasets is the mean–variance relationship, which describes the observed correlation between average gene expression and gene variance [18]. Because co-expression correlation aims to identify biologically relevant covariance relationships that are not due to technical factors, it is important to remove the mean–variance trend. Therefore, we transformed the matrix using the variance-stabilizing transform [18], as is recommended by the authors of the prominent gene co-expression correlation network analysis package “WGCNA” [58].

### Choice of Pearson correlation

Several correlation approaches were considered, including Spearman and Pearson. A large body of discussion is available regarding the strengths and weaknesses of each for various applications [59, 60]. In general, Pearson correlation is designed to observe linear relationships, whereas Spearman correlation is designed to observe any monotonic relationships [60]. Because of this, Pearson is a useful tool for finding genes which are covariant, providing a biologically interpretable understanding of their relationship. Relationships which are monotonic and non-linear, while potentially meaningful, are often difficult to interpret because of the wide range of patterns which can constitute monotonic correlation. Additionally, Pearson correlation is already implemented as a standard approach in gene co-expression studies [61–65], making results widely comparable. In particular, Pearson correlation is the basis for similar tools such as COXPRESdb [24], ARCHS4 [14], and GeneFriends [11].

Using an empirical approach that incorporates prior knowledge about gene–gene relationships, we tested the performance of both Pearson and Spearman correlation methods (Additional file 1: Figure S6). Because the purpose of Correlation AnalyzeR is to predict functional relationships, we tested whether Pearson or Spearman methods were better capable of predicting relationships between genes that belong to the same gene set within the MSigDB “Hallmark” collection [20]. We found that the correlation metrics typically agreed, but that there was a significantly greater correlation estimation by Pearson compared to Spearman (Additional file 1: Figure S6), indicating the better sensitivity of Pearson correlation for these biologically meaningful relationships. Additionally, when examining the gene pairs which showed the greatest specificity for Pearson or Spearman correlation, it was found that there was a noticeable tendency for the Spearman method to under-estimate tissue-specific relationships and to over-estimate non-linear relationships which defy simple interpretation. This pattern was exemplified by the top Pearson-specific gene pair (Additional file 1: Figure S7A), *MYL3*-*TCAP* (selected from the “HALLMARK_MYOGENESIS” gene set). Considering the specificity of this gene pair for muscle tissue, it was unsurprising to see that the strongest expression of both genes occurred in samples with “muscle” and “cardiac” tissue labels (Additional file 1: Figure S7A). However, while the Pearson correlation for this pair was 0.776, the Spearman correlation was only 0.189, exemplifying the sensitivity of the Pearson method to tissue-specific gene relationships. Conversely, the top Spearman-specific gene pair (Additional file 1: Figure S7B) was *TFF2*-*IL12B* from the “HALLMARK_KRAS_SIGNALING_DN” gene set, a set of genes which are downregulated by KRAS signaling. While the Spearman correlation was calculated to be 0.323, visual inspection of the plot indicates that there is not a discernable relationship (Additional file 1: Figure S7B). The Pearson coefficient for that same gene pair was 0.085, indicating that the Pearson method was more specific in this case. Taken together, these findings indicate the suitability of Pearson correlation in this context.

### Comparison with existing datasets

To ensure the validity of the co-expression results generated by *Correlation AnalyzeR*, genome-wide correlations for several genes (*BRCA1*, *AURKB*, and *HSP90AA1*) were calculated across all tissue and disease types. The top 500 co-expressed genes for each were compared to a large database of protein interactors (BioGRID) [26]. The overlap of co-expressed genes and protein interactors was highly significant in each case (Additional file 1: Figure S8A, S8C, and S8E). Furthermore, the genome-wide distribution of co-expression correlations for each gene was also compared with existing co-expression databases COXPRESdb [24], ARCHS4 [14], and GeneFriends [11]. As expected, a high degree of similarity was observed (Additional file 1: Figure S8B, S8D, and S8F). These results indicate the congruency of *Correlation AnalyzeR* co-expression correlations with both protein interactome data and independent co-expression databases.

### Limitations

While Correlation AnalyzeR provides multiple useful tools for inferring functional relationships from gene co-expression data, it is limited by several methodological and usability issues that will be addressed in future development.

One of the primary methodological limitations of this approach is the use of “flat” labels for tissue and disease types. While these labels provide a measure of convenience and allow the use of pre-calculated co-expression matrices, they cannot resolve the important biological relationships which are only revealed within subpopulations of samples. For example, “Breast Cancer” is a broad category which contains important subtypes, such as “triple-negative breast cancer”. A superior approach may be to implement a hierarchical tissue labeling scheme with automated annotation, such as MetaSRA [66]. This would allow for the characterization of co-expression relationships at multiple levels, from whole organs to specific cellular states. While challenging to implement due to the computational limitations inherent in calculating and storing co-expression matrices, it is still a promising future direction of development.

Another limitation of this approach is the use of Pearson correlation alone for exploring gene relationships. While linear correlations are beneficial from the standpoint of interpretation, there are additional valid approaches that show robust consistency and interpretability, such as biweight midcorrelation, Kendall, and Hoeffding’s D measure [58, 67]. While difficult to implement currently, a future direction of development for Correlation AnalyzeR will be to incorporate results from these correlation methods as well.

Another limitation of note is the “mean-correlation relationship”, a phenomena described in a recent preprint by *Wang *et al*.,* which refers to the tendency of gene co-expression correlation values to grow with the mean expression of the two genes under consideration [68]. This phenomenon represents a significant challenge for all co-expression studies and could introduce a systemic bias into the corGSEA method because it is reliant on ranking genes by correlation values. Further development of Correlation AnalyzeR will focus on testing and mitigating this trend by implementing normalization approaches, such as “Spatial Quantile Normalization” [68], which are designed to remove it.

Finally, one of the primary emerging applications for co-expression correlation analysis is single-cell RNA-Sequencing [69, 70]. Currently, Correlation AnalyzeR explicitly removes all single cell datasets from consideration due to concerns regarding the suitability of Pearson correlation in sparse data [16]. A primary future direction for Correlation AnalyzeR will be to incorporate methods for single-cell co-expression inference using emerging methods designed for that purpose [71, 72].

## Conclusion

Few gene co-expression correlation databases exist. Those currently available utilize a limited range of computational approaches for gene function prediction and, with the exception of GIANT, do not consider the impact of tissue and disease on co-expression. Furthermore, none exist which consider the genome-wide distribution of correlation values or provide gene comparison and gene list topology analysis. By implementing these methods, *Correlation AnalyzeR* provides users the opportunity to perform exploratory analyses of poorly characterized genes and uncover novel gene relationships. Additionally, current methods do not typically provide user-friendly summary tables or figures, necessitating the user possess some degree of bioinformatics skill. Correlation AnalyzeR provides high quality summary figures and tables within a flexible and user-friendly interface. These features were exemplified by our analysis of *BRCA1-NRF2* interplay in the context of bone cancers. In summary, Correlation AnalyzeR is a user-friendly database and web application which can reveal new gene functionalities and support the generation of novel biological hypotheses.

## Supplementary Information

**Additional file 1:** Supplementary figures.

**Additional file 2: **Table S1: Manifest of GEO samples used in Correlation AnalyzeR.

**Additional file 3:** Table S2: Comparison of Correlation AnalyzeR with similar tools.

## Data Availability

The dataset(s) supporting the conclusions of this article is(are) available in the ARCHS4 (human_matrix.h5, v8, Feb. 2020) repository, https://amp.pharm.mssm.edu/archs4/download.html [14]. We provide here a table of all the samples used in the final implementation of this tool, along with GEO metadata and assigned tissue and disease labels (Additional file 2: Table S1). Correlation AnalyzeR is available at https://correlationanalyzer.bishop-lab.com/ or as a standalone R-package at https://github.com/Bishop-Laboratory/correlationAnalyzeR. Additionally, custom scripts for generating figures in this manuscript are provided in the *misc/* folder within the R package repository. Finally, the source code for the web application is also provided publicly at https://github.com/Bishop-Laboratory/correlationAnalyzeR-ShinyApp.

## Abbreviations

GEO: Gene expression omnibus
SQL: Structured query language
Regex: Regular expression
VST: Variance-stabilizing transform
MSigDB: Molecular signatures database
GSEA: Gene set enrichment analysis
corGSEA: Correlation-ranked GSEA
NES: Normalized Enrichment Score
PCA: Principal component analysis
TSNE: T-distributed stochastic neighbor embedding
BRCA1: BRCA1 DNA repair associated
BRCA2: BRCA2 DNA repair associated
PARP: Poly (ADP-ribose) polymerase
FANCI: Fanconi Anemia Complementation Group I
EGFR: Epidermal growth factor receptor
E2F: E2F Transcription factor family
NQO1: NAD(P)H Quinone dehydrogenase 1
NRF2/NFE2L2: Nuclear factor erythroid 2-related factor 2

## References

[1] Zwick M, Kraemer O, Carter AJ. Dataset of the frequency patterns of publications annotated to human protein-coding genes, their protein products and genetic relevance. Data Br. 2019;25:104284. 10.1016/j.dib.2019.104284 PMC6702404 31453287

[2] Maertens A, Tran VH, Maertens M, Kleensang A, Luechtefeld TH, Hartung T, et al. Functionally enigmatic genes in cancer: using TCGA data to map the limitations of annotations. Sci Rep. 2020;10. 10.1038/s41598-020-60456-x PMC7057977 32139709

[3] Sashida G, Iwama A. Multifaceted role of the polycomb-group gene EZH2 in hematological malignancies. Int J Hematol. 2017;105:23–30. 10.1007/s12185-016-2124-x. 27830540 10.1007/s12185-016-2124-x

[4] Kolberg L, Kerimov N, Peterson H, Alasoo K. Co-expression analysis reveals interpretable gene modules controlled by trans-acting genetic variants. Elife. 2020;9:e58705. 10.7554/eLife.58705. 32880574 10.7554/eLife.58705 PMC7470823

[5] Liao Q, Liu C, Yuan X, Kang S, Miao R, Xiao H, et al. Large-scale prediction of long non-coding RNA functions in a coding–non-coding gene co-expression network. Nucleic Acids Res. 2011;39:3864–78. 10.1093/nar/gkq1348. 21247874 10.1093/nar/gkq1348 PMC3089475

[6] Wang T, Zhang J, Huang K. Generalized gene co-expression analysis via subspace clustering using low-rank representation. BMC Bioinform. 2019;20:196. 10.1186/s12859-019-2733-5. 10.1186/s12859-019-2733-5 PMC6509871 31074376

[7] von Mering C, Huynen M, Jaeggi D, Schmidt S, Bork P, Snel B. STRING: A database of predicted functional associations between proteins. Nucleic Acids Res. 2003;31:258–61. 12519996 10.1093/nar/gkg034 PMC165481

[8] Wiles AM, Doderer M, Ruan J, Gu TT, Ravi D, Blackman B, et al. Building and analyzing protein interactome networks by cross-species comparisons. BMC Syst Biol. 2010;4:36. 20353594 10.1186/1752-0509-4-36 PMC2859380

[9] Zanotto-Filho A, Dashnamoorthy R, Loranc E, De Souza LHT, Moreira JCF, Suresh U, et al. Combined gene expression and RNAi screening to identify alkylation damage survival pathways from fly to human. PLoS ONE. 2016;11:e0153970. 27100653 10.1371/journal.pone.0153970 PMC4839732

[10] Obayashi T, Hayashi S, Shibaoka M, Saeki M, Ohta H, Kinoshita K. COXPRESdb: a database of coexpressed gene networks in mammals. Nucleic Acids Res. 2008;36 Database issue:D77–82 10.1093/nar/gkm840 PMC2238883 17932064

[11] van Dam S, Craig T, de Magalhães JP. GeneFriends: a human RNA-seq-based gene and transcript co-expression database. Nucleic Acids Res. 2015;43 Database issue:D1124–32 10.1093/nar/gku1042 PMC4383890 25361971

[12] Franz M, Rodriguez H, Lopes C, Zuberi K, Montojo J, Bader GD, et al. GeneMANIA update 2018. Nucleic Acids Res. 2018;46:W60–4. 29912392 10.1093/nar/gky311 PMC6030815

[13] Wong AK, Krishnan A, Troyanskaya OG. GIANT 2.0: Genome-scale integrated analysis of gene networks in tissues. Nucleic Acids Res. 2018;46:W65-70. 29800226 10.1093/nar/gky408 PMC6030827

[14] Lachmann A, Torre D, Keenan AB, Jagodnik KM, Lee HJ, Wang L, et al. Massive mining of publicly available RNA-seq data from human and mouse. Nat Commun. 2018;9. 10.1038/s41467-018-03751-6 PMC5893633 29636450

[15] Bairoch A. The cellosaurus, a cell-line knowledge resource. J Biomol Tech. 2018. 10.7171/jbt.18-2902-002 PMC5945021 29805321

[16] Chen S, Mar JC. Evaluating methods of inferring gene regulatory networks highlights their lack of performance for single cell gene expression data. BMC Bioinform. 2018. 10.1186/s12859-018-2217-z PMC6006753 29914350

[17] Ballouz S, Verleyen W, Gillis J. Guidance for RNA-seq co-expression network construction and analysis: safety in numbers. Bioinformatics. 2015. 10.1093/bioinformatics/btv118 25717192

[18] Love MI, Huber W, Anders S. Moderated estimation of fold change and dispersion for RNA-seq data with DESeq2. Genome Biol. 2014. 10.1186/s13059-014-0550-8 PMC4302049 25516281

[19] Langfelder P, Horvath S. WGCNA: An R package for weighted correlation network analysis. BMC Bioinform. 2008. 10.1186/1471-2105-9-559 PMC2631488 19114008

[20] Liberzon A, Birger C, Thorvaldsdóttir H, Ghandi M, Mesirov JP, Tamayo P. The molecular signatures database hallmark gene set collection. Cell Syst. 2015. 10.1016/j.cels.2015.12.004 PMC4707969 26771021

[21] Subramanian A, Tamayo P, Mootha VK, Mukherjee S, Ebert BL, Gillette MA, et al. Gene set enrichment analysis: a knowledge-based approach for interpreting genome-wide expression profiles. Proc Natl Acad Sci USA. 2005. 10.1073/pnas.0506580102 PMC1239896 16199517

[22] Liberzon A, Subramanian A, Pinchback R, Thorvaldsdóttir H, Tamayo P, Mesirov JP. Molecular signatures database (MSigDB) 3.0. Bioinformatics. 2011. 10.1093/bioinformatics/btr260 PMC3106198 21546393

[23] Dolgalev I. msigdbr: MSigDB Gene sets for multiple organisms in a tidy data format. 2018. https://cran.r-project.org/package=msigdbr.

[24] Obayashi T, Kagaya Y, Aoki Y, Tadaka S, Kinoshita K. COXPRESdb v7: a gene coexpression database for 11 animal species supported by 23 coexpression platforms for technical evaluation and evolutionary inference. Nucleic Acids Res. 2019. 10.1093/nar/gky1155 PMC6324053 30462320

[25] Peterson BG, Carl P. Performance analytics: econometric tools for performance and risk analysis. 2019. https://cran.r-project.org/package=PerformanceAnalytics.

[26] Oughtred R, Stark C, Breitkreutz BJ, Rust J, Boucher L, Chang C, et al. The BioGRID interaction database: 2019 update. Nucleic Acids Res. 2019. 10.1093/nar/gky1079 PMC6324058 30476227

[27] Chen H. VennDiagram: generate high-resolution venn and euler plots. 2018. https://cran.r-project.org/package=VennDiagram.

[28] R Core Team. R: A Language and environment for statistical computing. 2019. https://www.r-project.org/.

[29] Chang W, Cheng J, Allaire J, Xie Y, McPherson J. shiny: Web application framework for R. 2019. https://cran.r-project.org/web/packages/shiny/index.html.

[30] Sergushichev AA. An algorithm for fast preranked gene set enrichment analysis using cumulative statistic calculation. bioRxiv. 2016.

[31] Bengtsson H. matrixStats: functions that apply to rows and columns of matrices (and to vectors). 2019. https://cran.rstudio.com/web/packages/matrixStats/index.html.

[32] Canty A, Ripley B. boot: Bootstrap functions (originally by angelo canty for S). 2019. https://cran.r-project.org/web/packages/boot/index.html.

[33] Krijthe J, Maaten L van der. Rtsne: T-Distributed stochastic neighbor embedding using a barnes-hut implementation. 2018. https://cran.r-project.org/web/packages/Rtsne/index.html.

[34] Yu G, Wang LG, Han Y, He QY. ClusterProfiler: an R package for comparing biological themes among gene clusters. Omi A J Integr Biol. 2012. 10.1089/omi.2011.0118 PMC3339379 22455463

[35] Kassambara A. ggpubr: “ggplot2” based publication ready plots. 2019. https://cran.r-project.org/web/packages/ggpubr/index.html.

[36] Wickham H. ggplot2: elegant graphics for data analysis. Springer-Verlag New York; 2016. https://ggplot2.tidyverse.org.

[37] Kolde R. pheatmap: Pretty Heatmaps. 2019. https://cran.r-project.org/package=pheatmap.

[38] Galili T, O’Callaghan A, Sidi J, et al. heatmaply: an R package for creating interactive cluster heatmaps for online publishing. Bioinformatics. 2017. 10.1093/bioinformatics/btx657. 10.1093/bioinformatics/btx657 PMC5925766 29069305

[39] Sievert C. plotly for R. 2018. https://plotly-r.com.

[40] Xie Y, Cheng J, Tan X. DT: A wrapper of the JavaScript library “DataTables.” 2019. https://cran.r-project.org/package=DT.

[41] Takaoka M, Miki Y. BRCA1 gene: function and deficiency. Int J Clin Oncol. 2018;:36–44. 10.1007/s10147-017-1182-2 28884397

[42] Gorthi A, Romero JC, Loranc E, Cao L, Lawrence LA, Goodale E, et al. EWS-FLI1 increases transcription to cause R-Loops and block BRCA1 repair in Ewing sarcoma. Nature. 2018. 10.1038/nature25748 PMC6318124 29513652

[43] Engert F, Kovac M, Baumhoer D, Nathrath M, Fulda S. Osteosarcoma cells with genetic signatures of BRCAness are susceptible to the PARP inhibitor talazoparib alone or in combination with chemotherapeutics. Oncotarget. 2017;8:48794–806. 27447864 10.18632/oncotarget.10720 PMC5564725

[44] Kovac M, Blattmann C, Ribi S, Smida J, Mueller NS, Engert F, et al. Exome sequencing of osteosarcoma reveals mutation signatures reminiscent of BRCA deficiency. Nat Commun. 2015;6:8940. 10.1038/ncomms9940. 26632267 10.1038/ncomms9940 PMC4686819

[45] Gorrini C, Baniasadi PS, Harris IS, Silvester J, Inoue S, Snow B, et al. BRCA1 interacts with Nrf2 to regulate antioxidant signaling and cell survival. J Exp Med. 2013;210:1529–44. 10.1084/jem.20121337. 23857982 10.1084/jem.20121337 PMC3727320

[46] Gowen LC, Johnson BL, Latour AM, Sulik KK, Koller BH. Brca1 deficiency results in early embryonic lethality characterized by neuroepithelial abnormalities. Nat Genet. 1996;12:191–4. 8563759 10.1038/ng0296-191

[47] Gudas JM, Li T, Nguyen H, Jensen D, Rauscher FJ 3rd, Cowan KH. Cell cycle regulation of BRCA1 messenger RNA in human breast epithelial cells. Cell growth Differ Mol Biol J Am Assoc Cancer Res. 1996;7:717–23. 8780885

[48] Miller HE, Gorthi A, Bassani N, Lawrence LA, Iskra BS, Bishop AJR. Reconstruction of Ewing Sarcoma developmental context from mass-scale transcriptomics reveals characteristics of EWSR1-FLI1 permissibility. Cancers (Basel). 2020;12:948. 10.3390/cancers12040948. 32290418 10.3390/cancers12040948 PMC7226175

[49] Wang A, Schneider-Broussard R, Kumar AP, MacLeod MC, Johnson DG. Regulation of BRCA1 expression by the Rb-E2F pathway. J Biol Chem. 2000. 10.1074/jbc.275.6.4532 10660629

[50] Kumaraswamy E, Wendt KL, Augustine LA, Stecklein SR, Sibala EC, Li D, et al. BRCA1 regulation of epidermal growth factor receptor (EGFR) expression in human breast cancer cells involves microRNA-146a and is critical for its tumor suppressor function. Oncogene. 2015. 10.1038/onc.2014.363 PMC4739738 25417703

[51] Ferreira BI, Alonso J, Carrillo J, Acquadro F, Largo C, Suela J, et al. Array CGH and gene-expression profiling reveals distinct genomic instability patterns associated with DNA repair and cell-cycle checkpoint pathways in Ewing’s sarcoma. Oncogene. 2008. 10.1038/sj.onc.1210845 17952124

[52] Shen J, Rasmussen M, Dong Q-R, Tepel M, Scholze A. Expression of the NRF2 target gene NQO1 is enhanced in mononuclear cells in human chronic kidney disease. Oxid Med Cell Longev. 2017;2017:9091879. 28785379 10.1155/2017/9091879 PMC5530440

[53] Nguyen T, Nioi P, Pickett CB. The Nrf2-antioxidant response element signaling pathway and its activation by oxidative stress. J Biol Chem. 2009;284:13291–5. 19182219 10.1074/jbc.R900010200 PMC2679427

[54] Wang X-J, Sun Z, Villeneuve NF, Zhang S, Zhao F, Li Y, et al. Nrf2 enhances resistance of cancer cells to chemotherapeutic drugs, the dark side of Nrf2. Carcinogenesis. 2008;29:1235–43. 10.1093/carcin/bgn095. 18413364 10.1093/carcin/bgn095 PMC3312612

[55] Lignitto L, LeBoeuf SE, Homer H, Jiang S, Askenazi M, Karakousi TR, et al. Nrf2 activation promotes lung cancer metastasis by inhibiting the degradation of Bach1. Cell. 2019;178(316–329):e18. 10.1016/j.cell.2019.06.003 PMC6625921 31257023

[56] Ghandi M, Huang FW, Jané-Valbuena J, Kryukov GV, Lo CC, McDonald ER, et al. Next-generation characterization of the cancer cell line encyclopedia. Nature. 2019;569:503–8. 10.1038/s41586-019-1186-3. 31068700 10.1038/s41586-019-1186-3 PMC6697103

[57] King M-C, Marks JH, Mandell JB. Breast and Ovarian cancer risks due to inherited mutations in BRCA1; and BRCA2 Science (80-). 2003;302:643 LP–646. 10.1126/science.1088759. 10.1126/science.1088759 14576434

[58] Langfelder P, Horvarth S. WGCNA package FAQ. WGCNA: an R package for weighted correlation network analysis. 2017. https://horvath.genetics.ucla.edu/html/CoexpressionNetwork/Rpackages/WGCNA/faq.html. Accessed 10 Aug 2019. 10.1186/1471-2105-9-559 PMC2631488 19114008

[59] de Winter JCF, Gosling SD, Potter J. Comparing the Pearson and Spearman correlation coefficients across distributions and sample sizes: a tutorial using simulations and empirical data. Psychol Methods. 2016;21:273–90. 27213982 10.1037/met0000079

[60] Abdullah MB. On a robust correlation coefficient. J R Stat Soc Ser D (The Stat). 1990;39:455–60. 10.2307/2349088.

[61] Tang J, Kong D, Cui Q, Wang K, Zhang D, Gong Y, et al. Prognostic genes of breast cancer identified by gene co-expression network analysis. Front Oncol . 2018;8:374. https://www.frontiersin.org/article/. 10.3389/fonc.2018.00374 10.3389/fonc.2018.00374 PMC6141856 30254986

[62] Aoki K, Ogata Y, Shibata D. Approaches for extracting practical information from gene co-expression networks in plant biology. Plant Cell Physiol. 2007;48:381–90. 10.1093/pcp/pcm013. 17251202 10.1093/pcp/pcm013

[63] Jupiter D, Chen H, VanBuren V. STARNET 2: a web-based tool for accelerating discovery of gene regulatory networks using microarray co-expression data. BMC Bioinform. 2009;10:332. 10.1186/1471-2105-10-332 PMC2765977 19828039

[64] Li J, Zhou D, Qiu W, Shi Y, Yang J-J, Chen S, et al. Application of weighted gene co-expression network analysis for data from paired design. Sci Rep. 2018;8:622. 10.1038/s41598-017-18705-z. 29330528 10.1038/s41598-017-18705-z PMC5766625

[65] Li B, Pu K, Wu X. Identifying novel biomarkers in hepatocellular carcinoma by weighted gene co-expression network analysis. J Cell Biochem. 2019;120:11418–31. 10.1002/jcb.28420. 30746803 10.1002/jcb.28420

[66] Bernstein MN, Doan A, Dewey CN. MetaSRA: normalized human sample-specific metadata for the sequence read archive. Bioinformatics. 2017;33:2914–23. 28535296 10.1093/bioinformatics/btx334 PMC5870770

[67] Kumari S, Nie J, Chen H-S, Ma H, Stewart R, Li X, et al. Evaluation of gene association methods for coexpression network construction and biological knowledge discovery. PLoS ONE. 2012;7:e50411. 23226279 10.1371/journal.pone.0050411 PMC3511551

[68] Wang Y, Hicks SC, Hansen KD. Co-expression analysis is biased by a mean-correlation relationship. bioRxiv. 2020:2020. 10.1101/2020.02.13.944777.

[69] Bartlett TE, Müller S, Diaz A. Single-cell Co-expression Subnetwork Analysis. Sci Rep. 2017;7:15066. 10.1038/s41598-017-15525-z. 29118406 10.1038/s41598-017-15525-z PMC5678118

[70] Iacono G, Massoni-Badosa R, Heyn H. Single-cell transcriptomics unveils gene regulatory network plasticity. Genome Biol. 2019;20:110. 10.1186/s13059-019-1713-4. 31159854 10.1186/s13059-019-1713-4 PMC6547541

[71] van Dijk D, Sharma R, Nainys J, Yim K, Kathail P, Carr AJ, et al. Recovering gene interactions from single-cell data using data diffusion. Cell. 2018;174(716–729):e27. 10.1016/j.cell.2018.05.061 PMC6771278 29961576

[72] Gallivan CP, Ren H, Read EL. Analysis of single-cell gene pair coexpression landscapes by stochastic kinetic modeling reveals gene-pair interactions in development . Front Genet. 2020;10:1387. https://www.frontiersin.org/article/. 10.3389/fgene.2019.01387. 10.3389/fgene.2019.01387 PMC7005996 32082359

