# Supplementary material for: Correlation AnalyzeR: functional predictions from gene co-expression correlations
Source: BMC Bioinformatics. 2021 Apr 20;22:206. doi: 10.1186/s12859-021-04130-7 (PMC8056587; doi:10.1186/s12859-021-04130-7)
Supplement: Supplementary file 1 — Additional file 1: Supplementary figures. [file 12859_2021_4130_MOESM1_ESM.pdf]

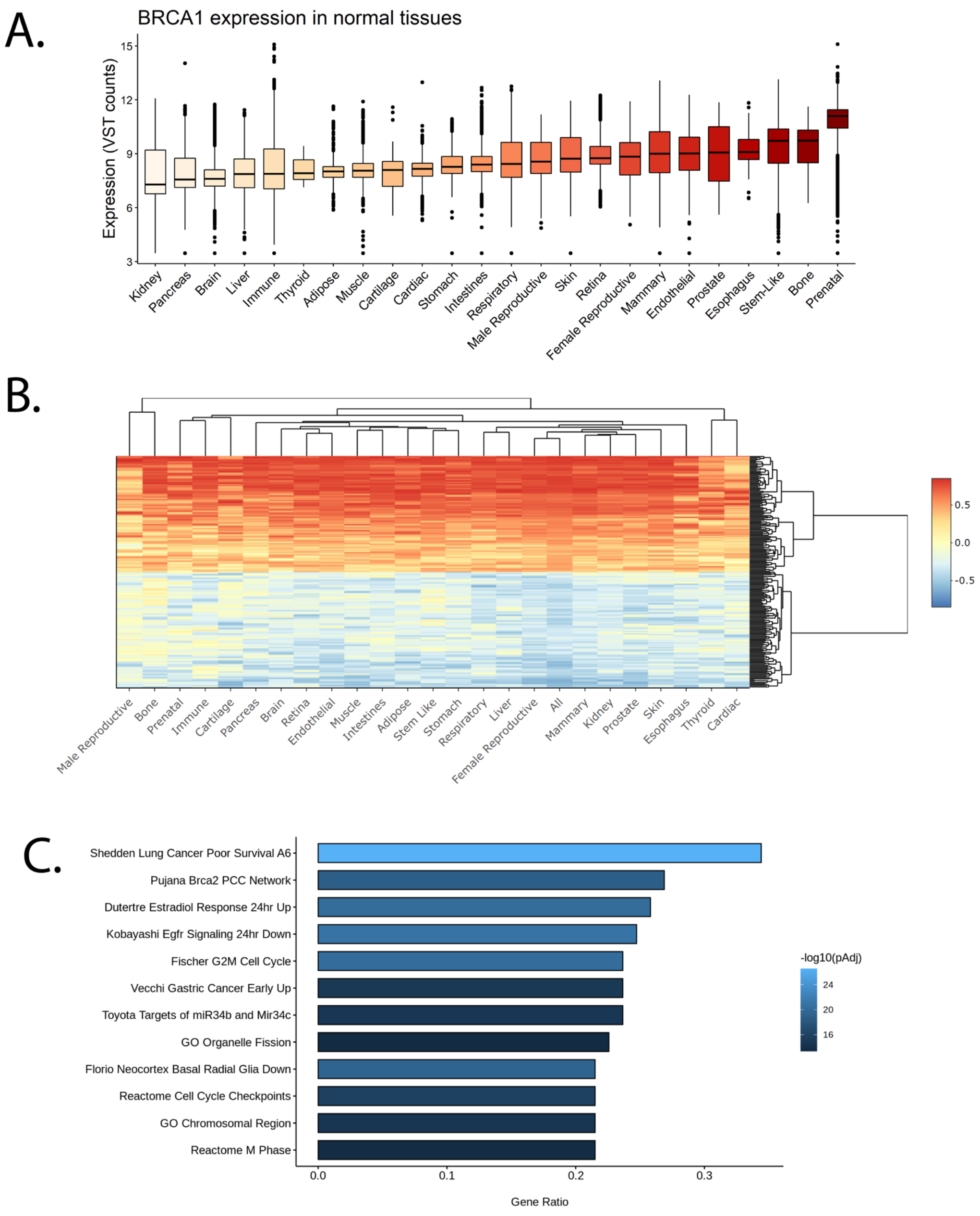

Figure S1: Group mode analysis in normal tissues within single gene mode for BRCA1. (A) The expression of BRCA1 across normal tissues in VST-normalized read counts. (B) The top 100 consistently co-correlated and anti-correlated genes with BRCA1 across normal tissues. Color bar represents Pearson correlation values. (C) The enrichment of GO biological process terms within the top 100 consistently co-correlated genes. Color bar represents adjusted p value of enrichment (Benjamini-Hochberg correction).

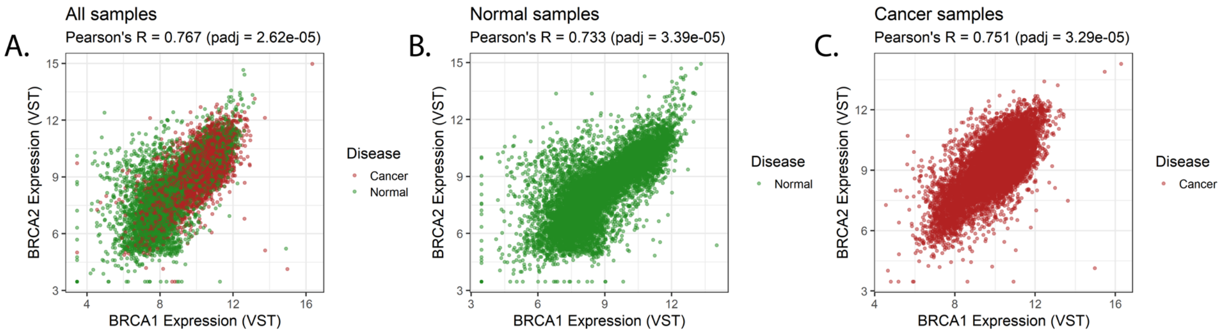

Figure S2: BRCA1 and BRCA2 gene co-expression correlation analysis in normal and cancer samples. (A) A scatter plot comparing BRCA1 and BRCA2 expression across all samples with disease condition colored. Pearson's R and a P adjusted value are reported from 'Holm' correction. (B) Same as A, but the analysis was limited only to Normal tissue samples. (C) Same as A, but the analysis was limited only to Cancer samples.

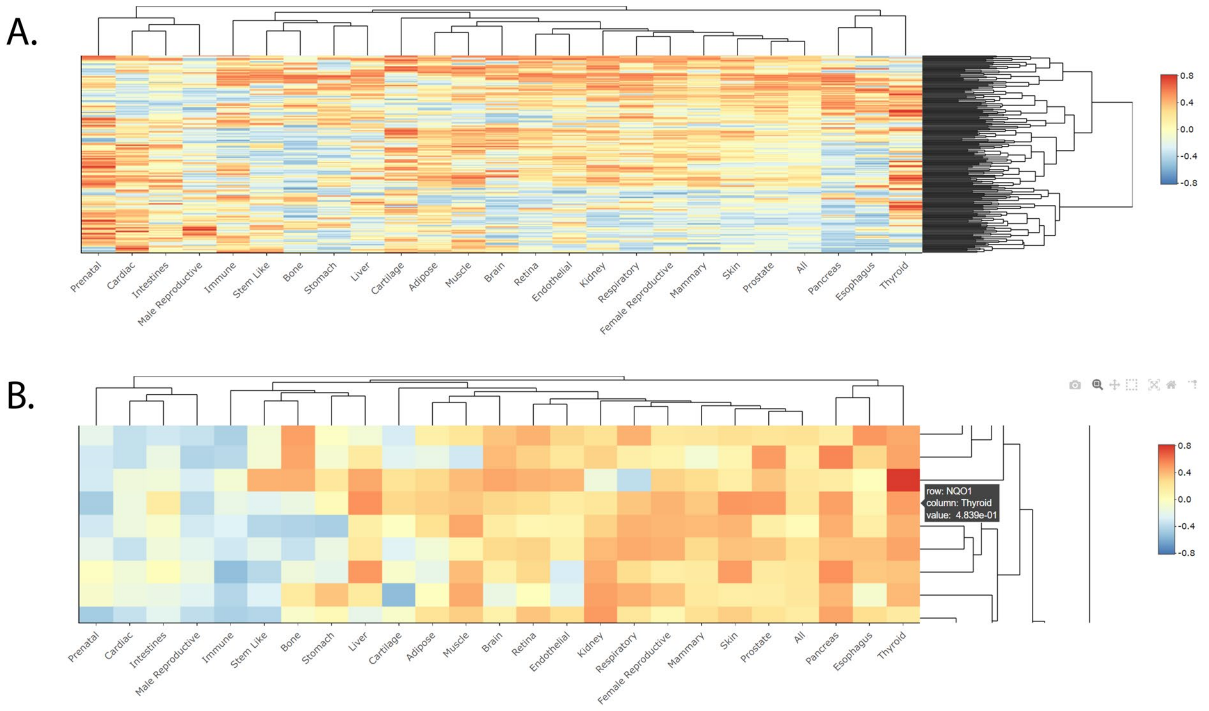

Figure S3: Group mode analysis in normal tissues within single gene mode for BRCA1 showing variable genes. (A) The top 100 differentially co-expressed genes with BRCA1 across normal tissues. (B) A close-up of (A) in which NQO1 co-expression with BRCA1 across normal tissues is highlighted. Color bar represents Pearson correlation values.

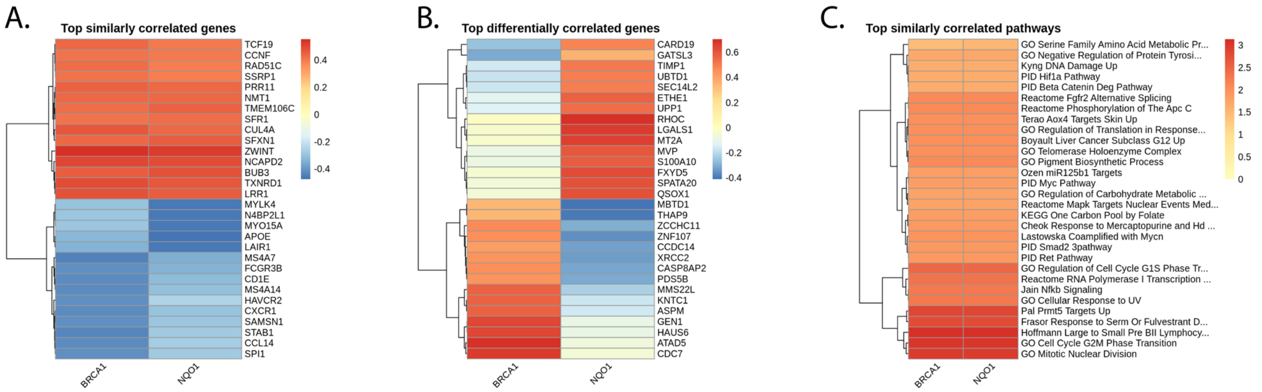

Figure S4: Analysis of BRCA1 and NQO1 with gene vs gene mode. (A) Heatmaps depict the top similarly and (B) differentially correlated genes between BRCA1 and NQO1 in bone cancer samples. (A-B) Color bar represents Pearson correlation values. (C) Heatmap showing similar corGSEA results for BRCA1 and NQO1 in bone cancer samples. Color bar represents normalized enrichment score.

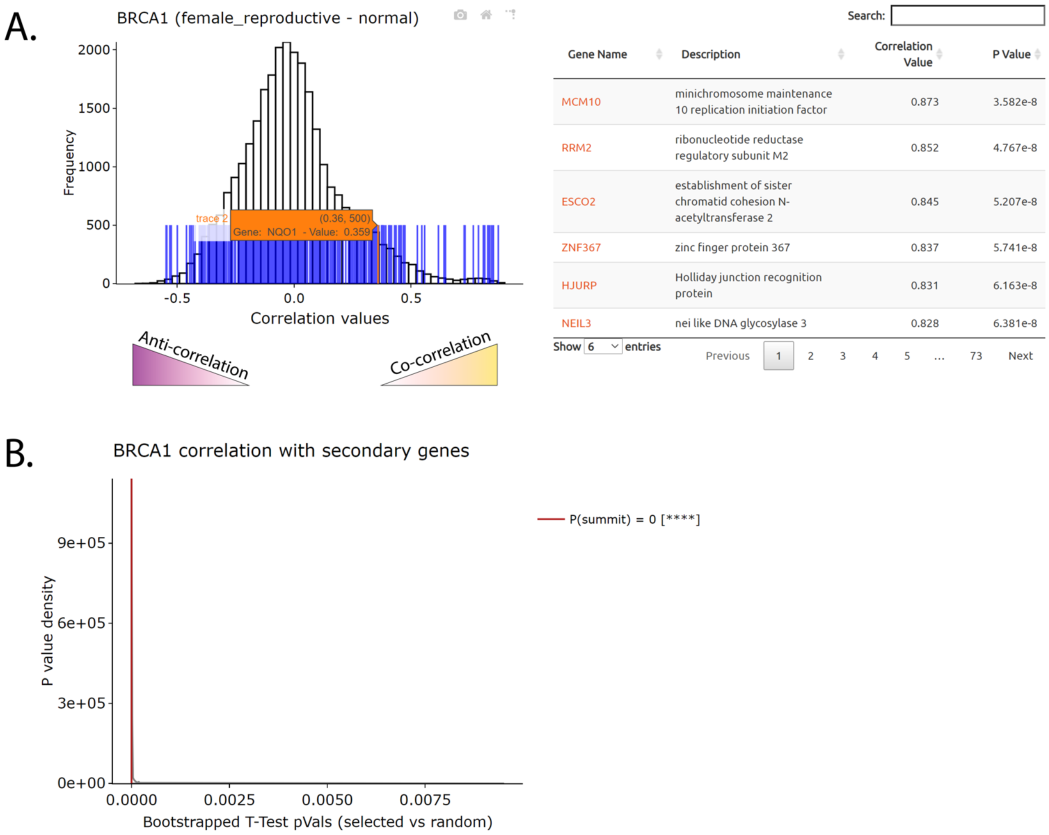

Figure S5: Analysis of BRCA1-NRF2 pathway interplay in normal female reproductive tissues (e.g., ovaries, cervix) using Gene vs Gene List mode. (A-B) Results of comparing BRCA1 and NRF2 gene targets ("NFE2L2.V2" MSigDB gene set). (A) Interactive histogram showing the location of the NRF2-pathway genes (blue bars overlaid on histogram on left) within the correlation value distribution of BRCA1 with linked data table (right panel). The position of NQO1 in the distribution is highlighted. P value determined from Pearson correlation. "Anti-correlation" and "Co-correlation" annotations not generated by Correlation AnalyzeR. (B) Density plot generated by permutation testing. The summit represents the empirically determined significance of BRCA1's correlation with the NRF2 pathway (labeled "secondary genes" in plot) (see methods).

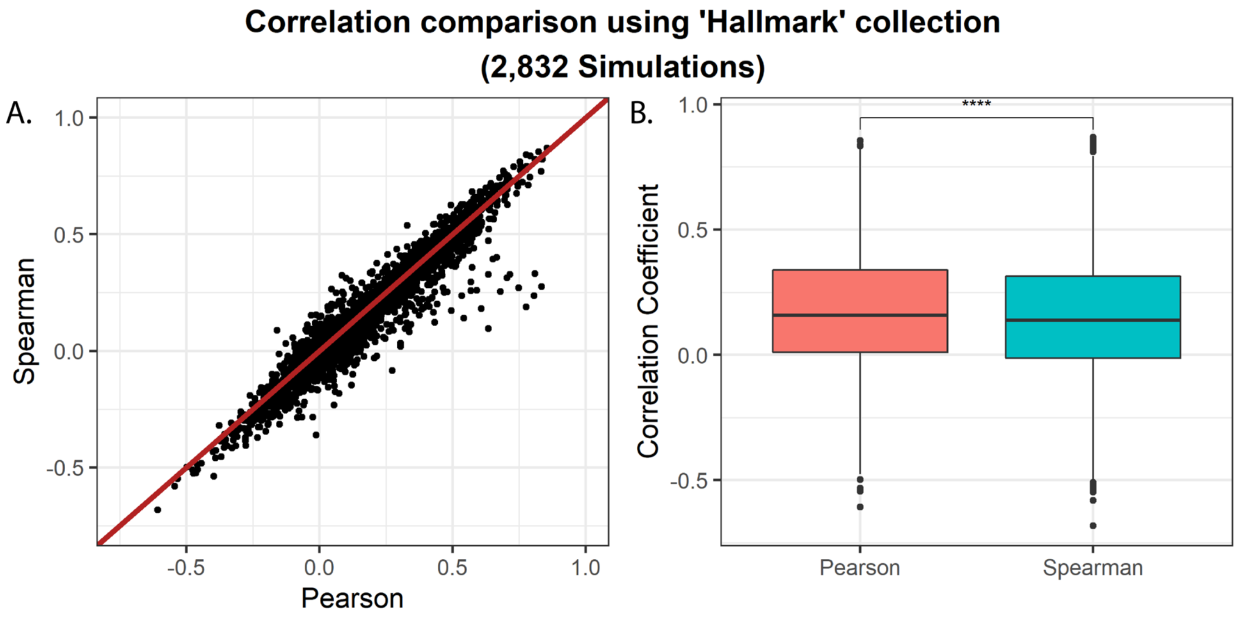

*Figure S6: Comparison of co-expression correlation results obtained from Pearson and Spearman methods within the "Hallmark" collection. Gene pairs from the same gene sets were randomly selected over 2,832 bootstrapped simulations and the Pearson and Spearman co-expression correlations were calculated. (A) Scatter plot showing the Spearman and Pearson correlation coefficients for each simulation. (B) Box plot comparing the mean Pearson and Spearman correlation coefficients. **** indicates one-tailed t-test p value < .0001; actual p = 7.503e-05.*

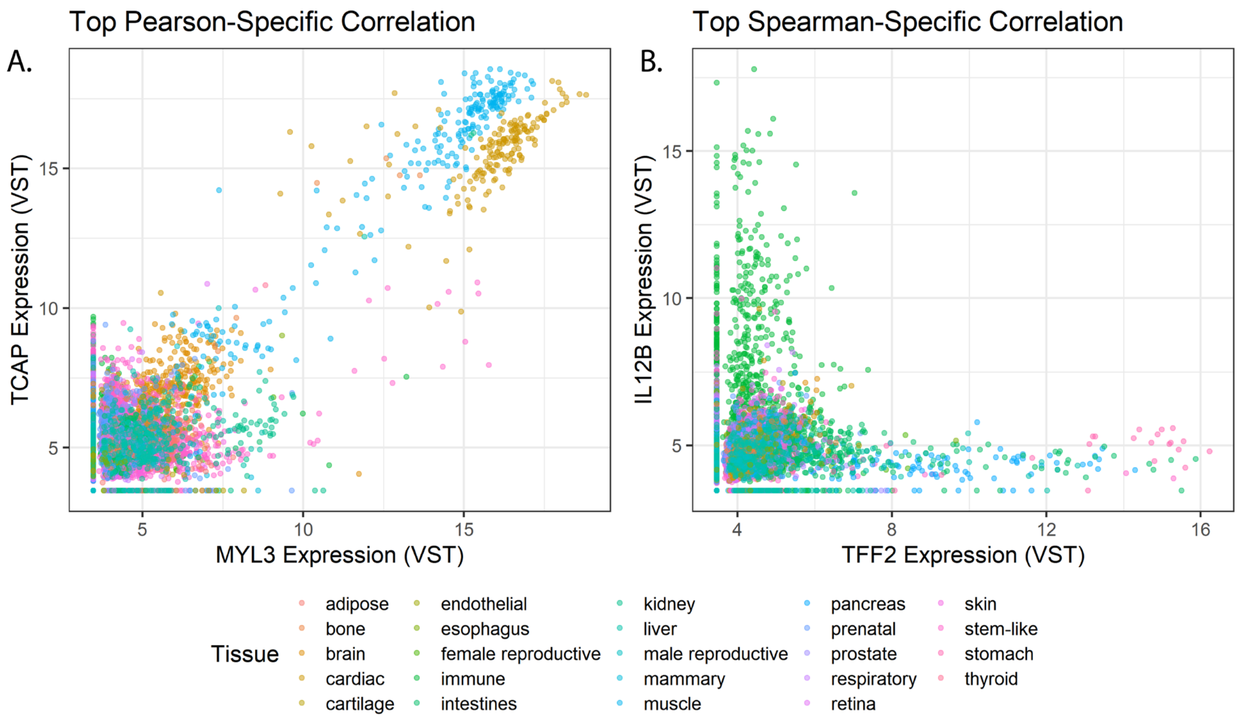

Figure S7: Top Pearson-specific and Spearman-specific correlations. From bootstrapping, the co-expression correlation Pearson and Spearman coefficients were determined for gene pairs belonging to the same "Hallmark" gene sets. The top Pearson-specific gene pairs and Spearman-specific gene pairs were determined by finding the largest correlation coefficient discrepancies between the two methods. (A) Scatter plot showing the top Pearson-specific gene correlation (MYL3 – TCAP). (B) Scatter plot showing the top Spearman-specific gene correlation (TFF2, IL12B).

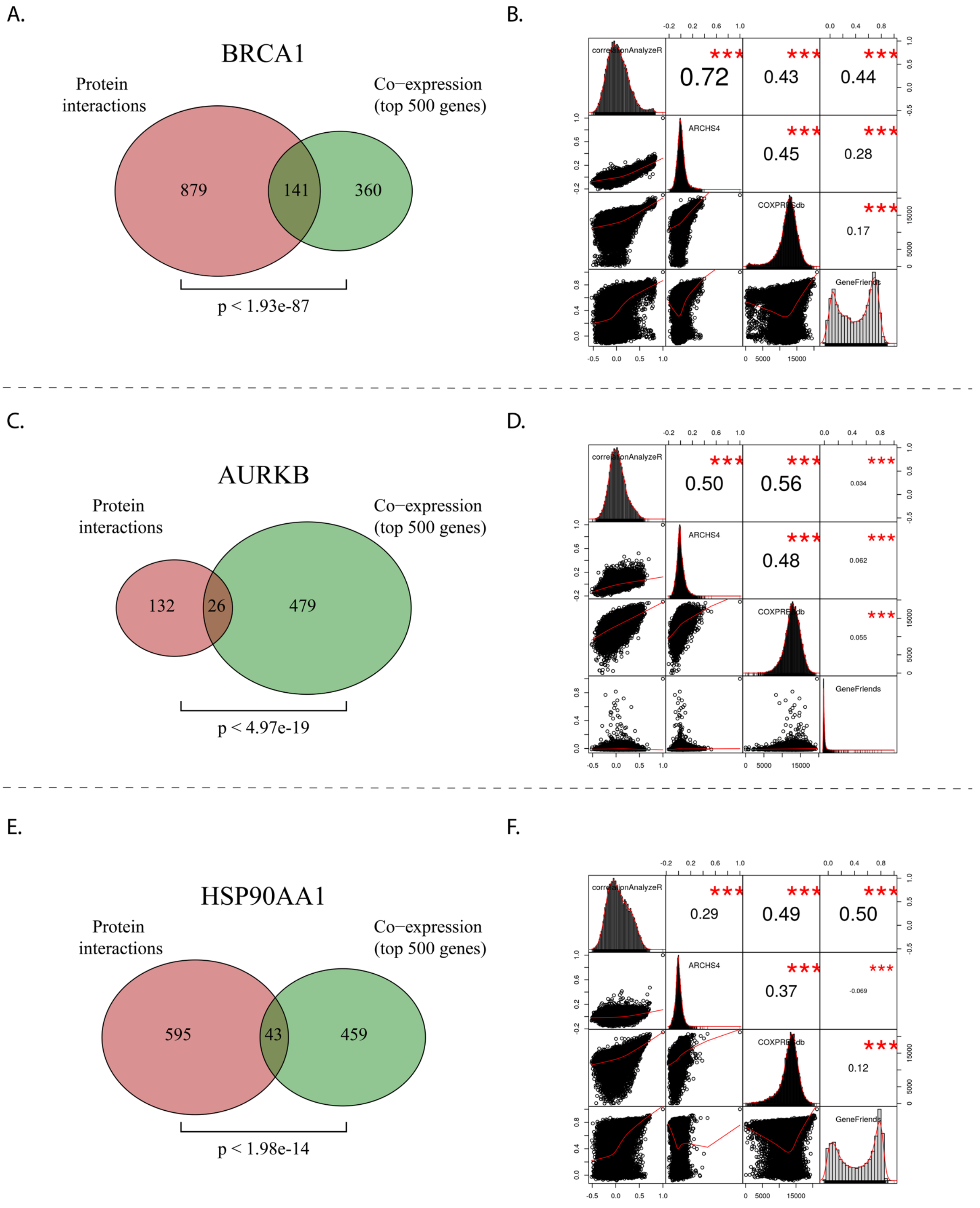

*Figure S8: Comparison of Correlation AnalyzeR co-expression correlations to public databases. The genes tested are (A-B) BRCA1, (C-D) AURKB, and (E-F) HSP90AA1. (A, C, E) Venn diagrams showing the top 500 gene correlations from Correlation AnalyzeR compared to the protein interactors for each gene in BioGRID. P value calculated via hypergeometric test. (B, D, F) Spearman correlation plots demonstrate the similarity between correlation values ascribed to each gene by correlationAnalyzeR, ARCHS4, GeneFriends, and COXPRESdb. *** Spearman p < .001.*
