# Supplementary material for: Correlation AnalyzeR: functional predictions from gene co-expression correlations
Source: BMC Bioinformatics. 2021 Apr 20;22:206. doi: 10.1186/s12859-021-04130-7 (PMC8056587; doi:10.1186/s12859-021-04130-7)
Supplement: Supplementary file 3 — Additional file 3: Table S2: Comparison of Correlation AnalyzeR with similar tools. [file 12859_2021_4130_MOESM3_ESM.pdf]

Table S2: Comparison matrix of available tools for functional prediction based on co-expression correlations

Tool	Functional Prediction Method	Tissue-specific	Disease-specific	Analysis Types	Results presentation	Implementation	Publication
**COXPRESdb**	Over-representation analysis (ORA) from top 50 co-expressed genes	No	No	Single gene prediction; Gene comparison	Static results tables with minimal support for static visualizations	Web application; API	Obayashi et al. 2008 [10]; Obayashi et al. 2019 [24]
**GeneFriends**	ORA from top 5% of co-expressed genes	No	No	Single gene prediction	Static tables with an option to export a network to Biolayout	Web application	van Dam et al. 2015 [11]
**GeneMANIA**	ORA from co-expression and protein interaction network	No	No	Single gene prediction	Interactive network with summary tables	Web application; Cytoscape app	Franz et al. 2018 [12]
**GIANT**	ORA from co-expression, protein interaction, gene-set co-membership, and TF binding prediction	Yes	No	Single gene prediction; Gene comparison; Network comparison	Interactive network and heatmaps with summary tables	Web application; API	Wong et al. 2018 [13]
**ARCHS4**	Gene sets ranked by mean correlation of member genes	No	No	Single gene prediction	Summary tables of top enrichment results	Web application; API	Lachmann et al. 2018 [14]
**Correlation AnalyzeR**	GSEA of genome-wide correlation values (corGSEA)	Yes	Yes	Single gene prediction; Gene comparison; Gene vs Gene list comparison; Gene list topology	Summary tables with interactive gene-level and pathway-level plots	Web application; R package	This article.
